# Supplementary figures and images for: Definition of the immune evasion-replication interface of rabies virus P protein
Source: PLoS Pathog. 2021 Jul 8;17(7):e1009729. doi: 10.1371/journal.ppat.1009729 (PMC8291714; doi:10.1371/journal.ppat.1009729)

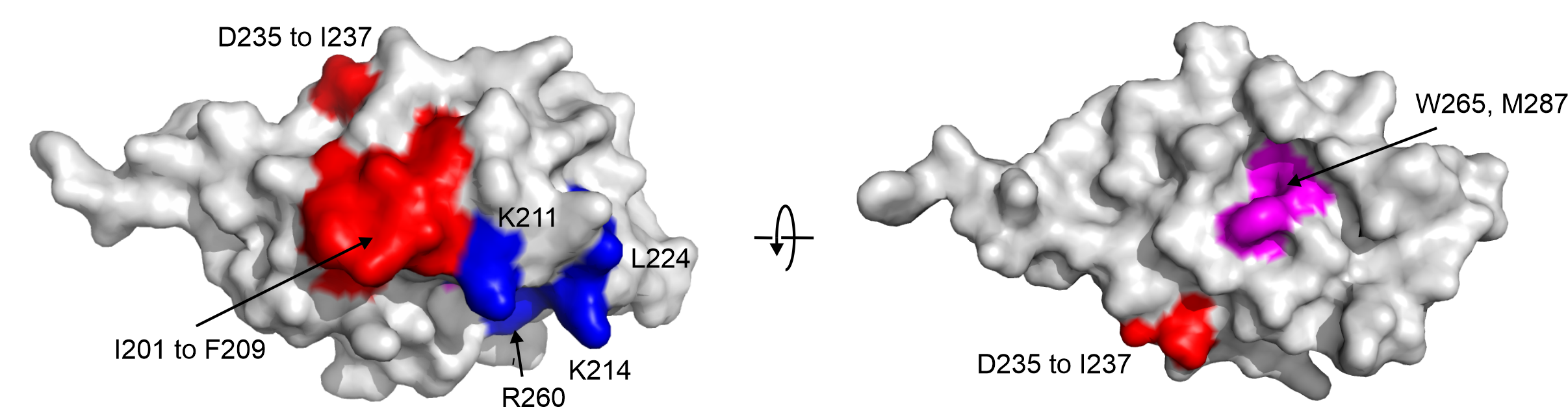

Supplement: S1 Fig — In red is the location of the STAT1 binding site (I201 to F209, D235 to I237) which is proximal to the positive patch (K211, K214, R260) and the hydrophobic residue L224 that bind the flexible peptide of the N protein (Npep) and characterized in this study, and shown in blue. The residues of the W-hole (W265 and M287) are on the opposite side of the protein and have been suggested as an additional N protein binding site. (TIF) [file ppat.1009729.s001.tif]

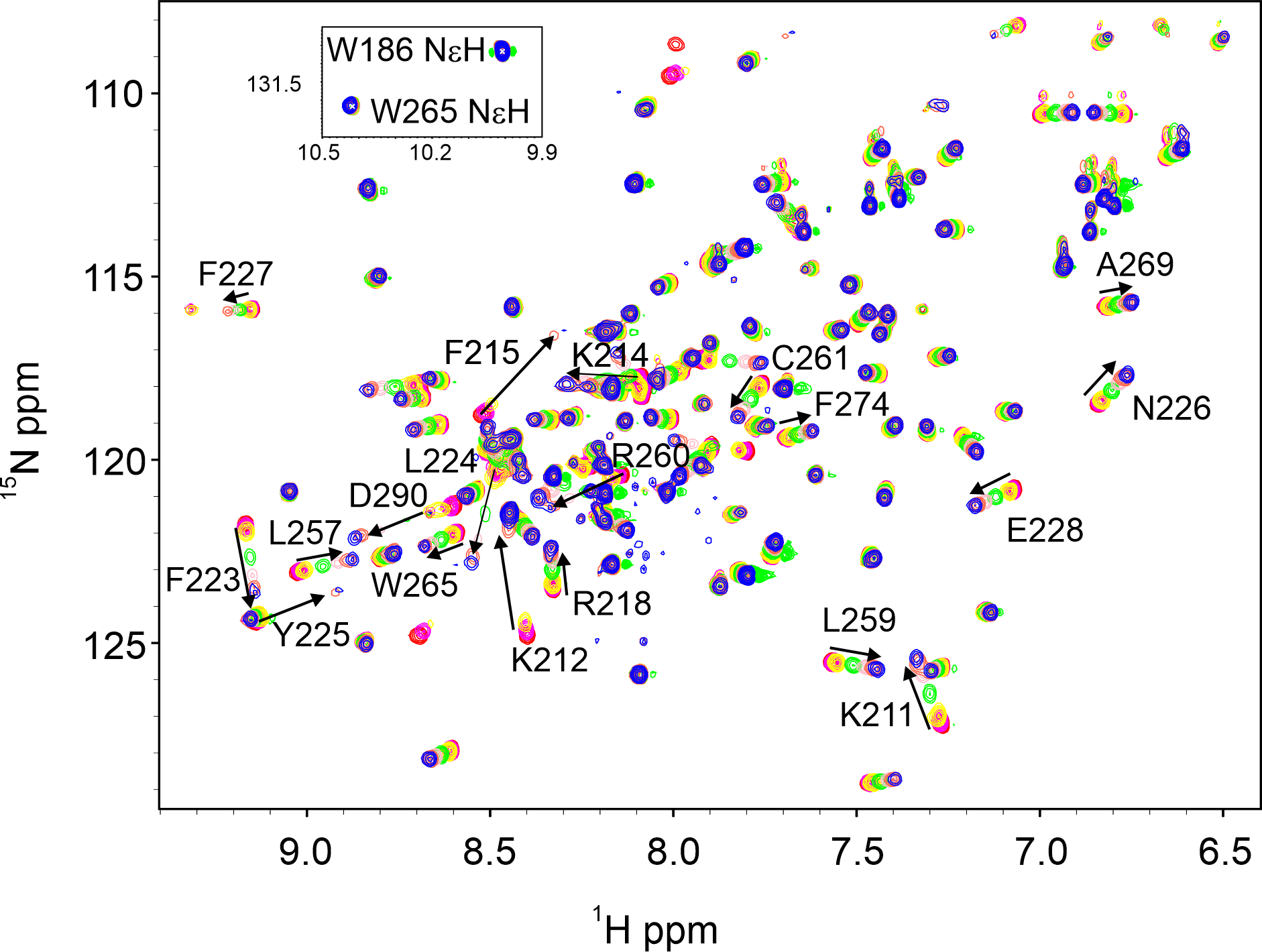

Supplement: S2 Fig — Titration of 100 μM PCTD with linear wild-type N-pep, 0 (red), 50 (magenta), 100 (yellow), 200 (green), 400 (pink), 800 (tomato), 1200 (blue) μM. Spectra were recorded at 25°C and pH 6.8. (TIF) [file ppat.1009729.s002.tif]

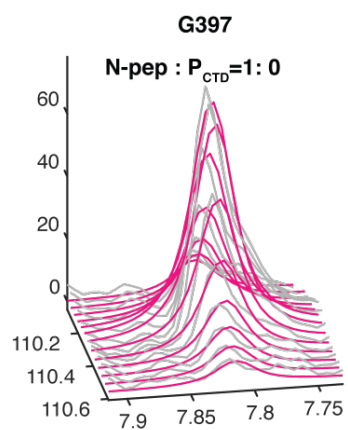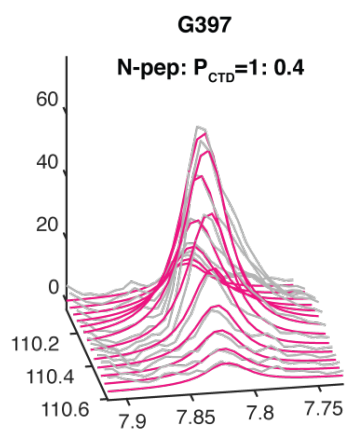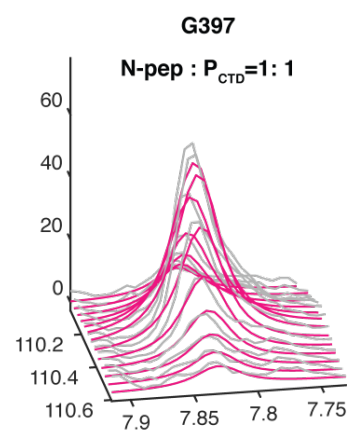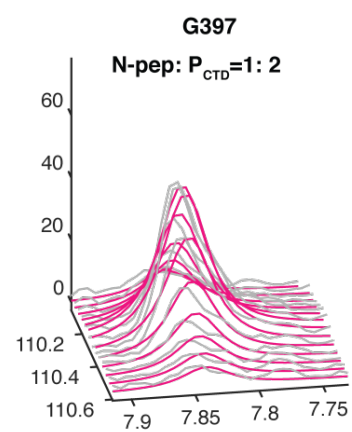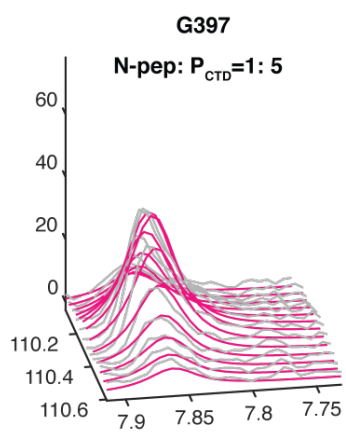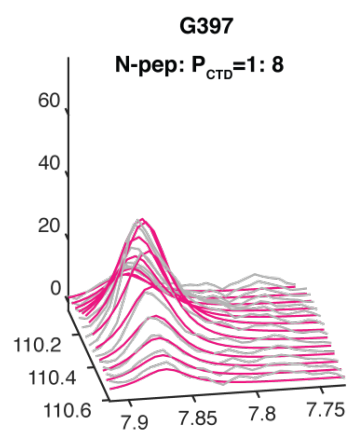

Supplement: S3 Fig — The lineshape of G397 (grey) of N-pep at apo state and 11 different titration points (5 shown here) were fitted into a two-state binding model (magenta) with a KD of 215 ± 6 μM and koff of 3164 ± 168 s-1. (PDF) [file ppat.1009729.s003.pdf]

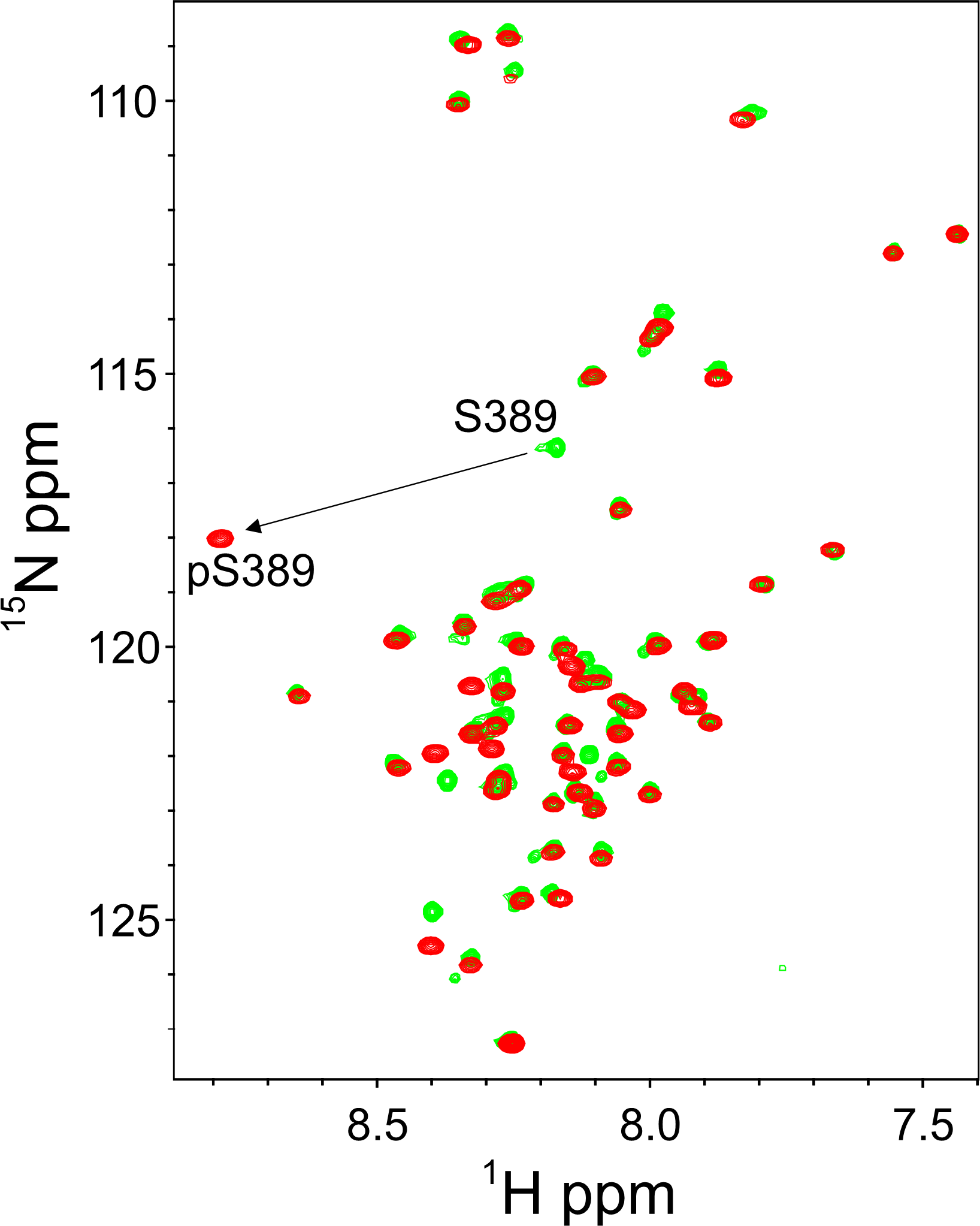

Supplement: S4 Fig — Spectra were recorded at 25°C and pH 6.8. Ser389 and phosphorylated S389 (pS389) were labelled in the spectra. (TIF) [file ppat.1009729.s004.tif]

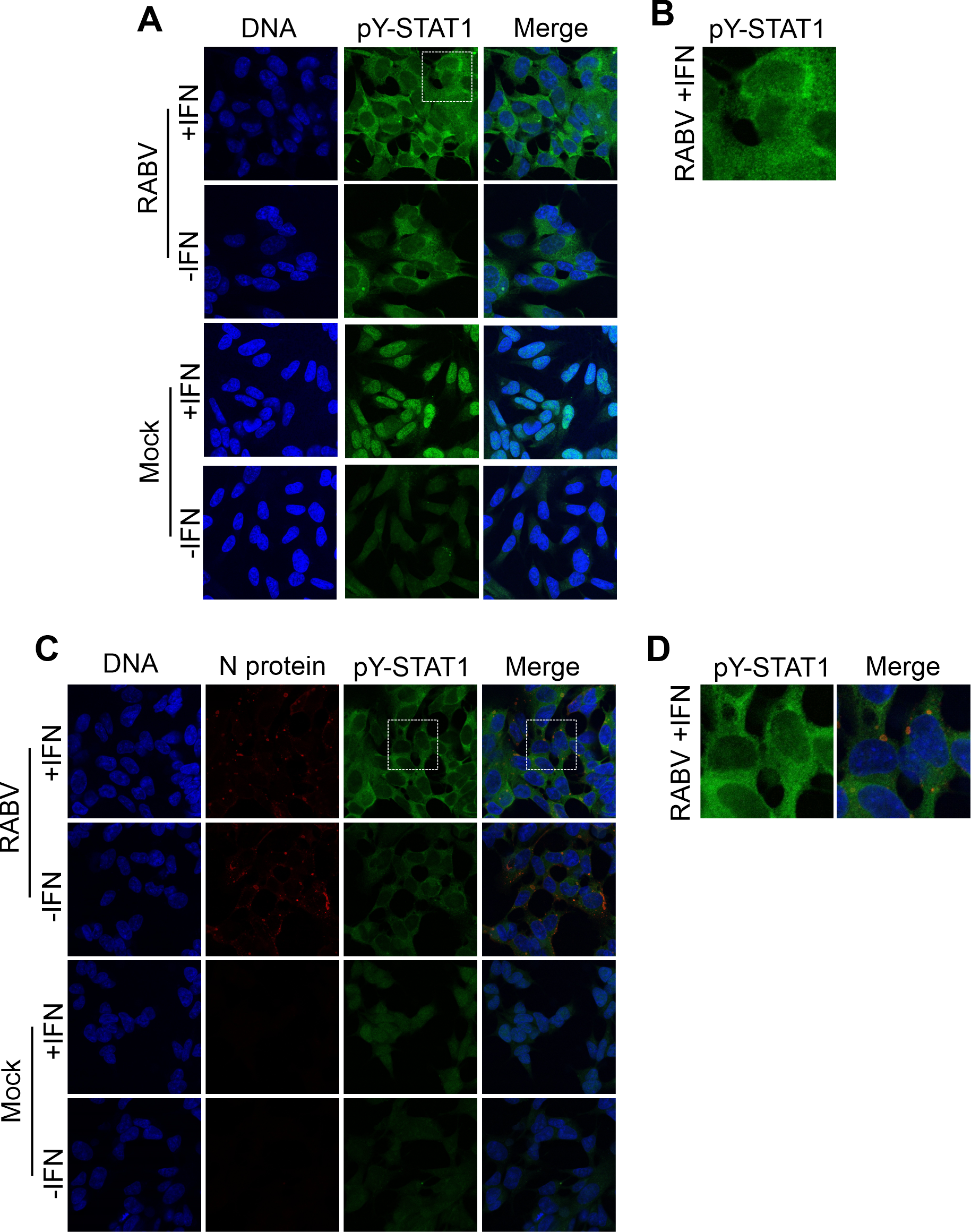

Supplement: S5 Fig — SK-N-SH cells were infected with CE-NiP RABV (MOI = 5, 24 h) before fixation and immunostaining for pY-STAT1 (A, B) or for both N protein and pY-STAT1 (C, D); Hoechst 33342 was used to localize nuclei (DNA). Regions within the dashed white box (A, C) are expanded in B and D. (TIF) [file ppat.1009729.s005.tif]

*
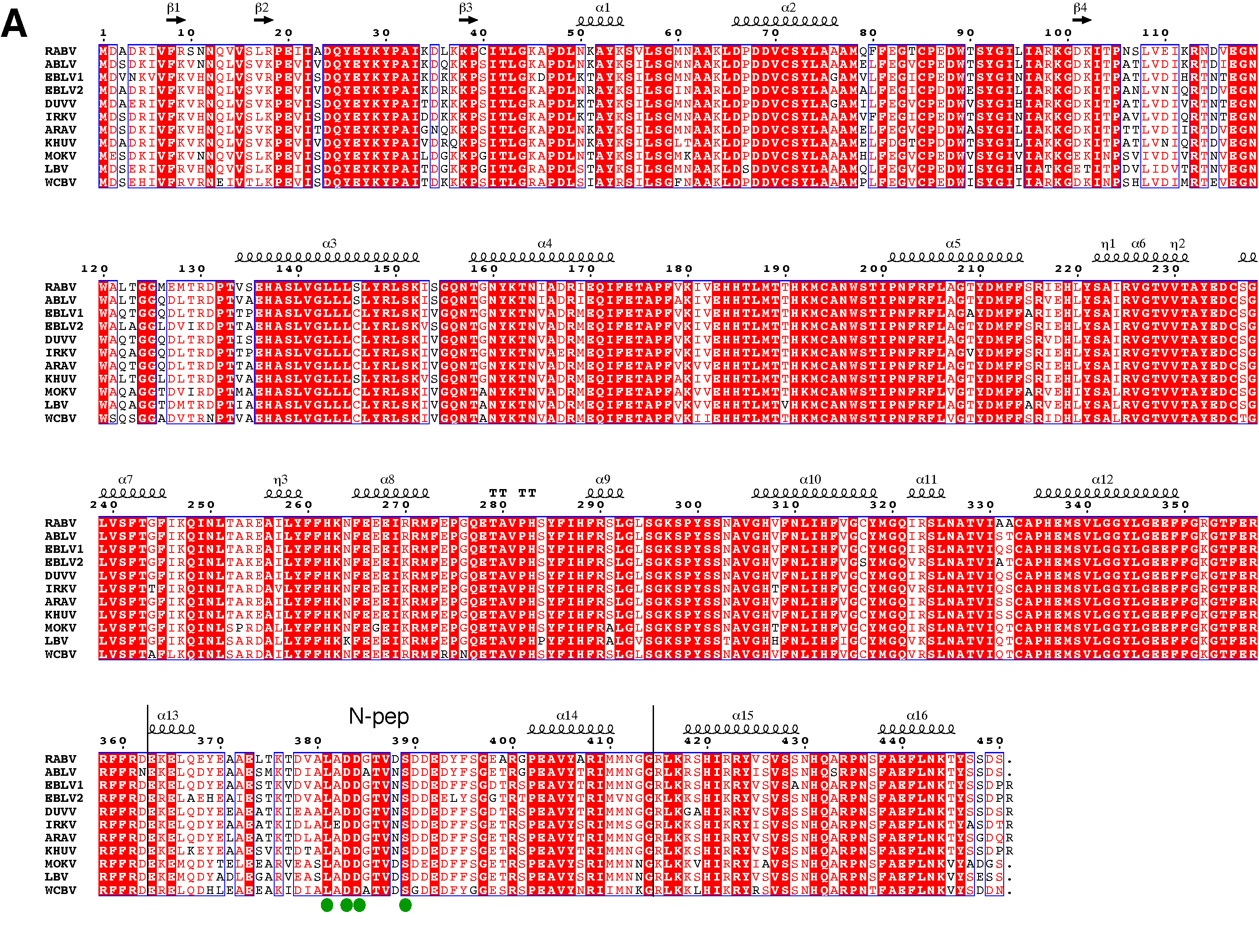

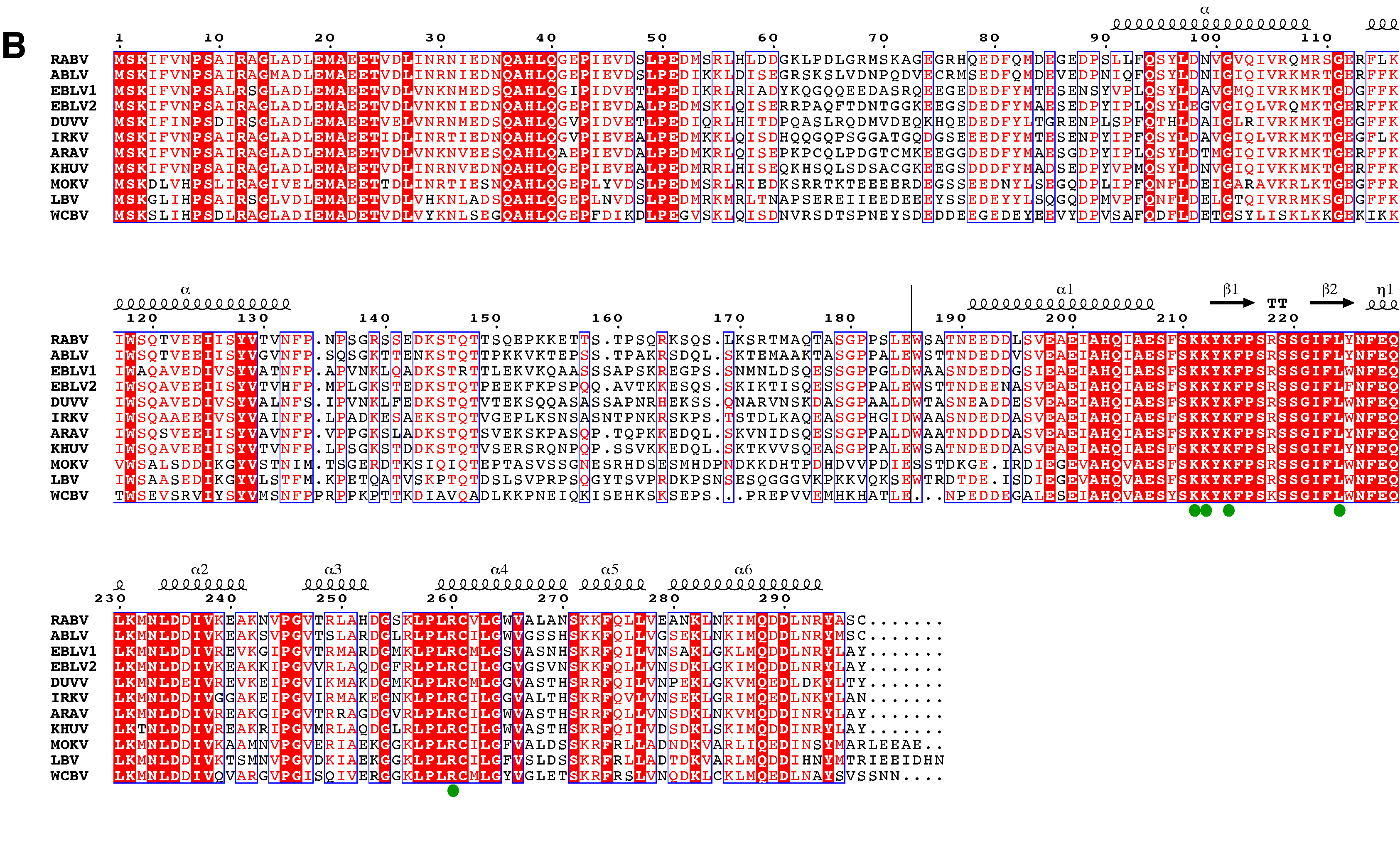
*

Supplement: S6 Fig — Multiple sequence alignment of Lyssavirus N (A) and P proteins (B). The residues critical for N-pep/PCTD interaction are marked with a green dot. Although P protein is much less conserved between lyssaviruses than N, the critical residues identified within the N/P interface appear to be highly conserved among the lyssaviruses. Protein sequences were retrieved from the GenBank: RABV (N: O55611, P: Q9IPJ8), ABLV (N: Q8JTH3, P: Q8JTH2), EBLV1 (N: A4UHP8, P: A4UHP9), EBLV2 (N: A4UHQ3, P: A4UHQ4), DUVV (N: Q66453, P: O56774), IRKV (N: Q5VKP6, P: Q5VKP5), ARAV (N: Q6X1D8, P: Q6X1D7), KHUV (N: Q6X1D4, P: Q6X1D3), MOKV (N: P0C570, P: P0C569), LBV (N: Q82994, P: O56773), WBCV (N: Q5VKP2, P: Q5VKP1); accession numbers are shown in parentheses. (DOCX) [file ppat.1009729.s006.docx]
