# Supplementary material for: Definition of the immune evasion-replication interface of rabies virus P protein
Source: PLoS Pathog. 2021 Jul 8;17(7):e1009729. doi: 10.1371/journal.ppat.1009729 (PMC8291714; doi:10.1371/journal.ppat.1009729)
Supplement: Attachment — Submitted filename: Response_to_reviewers.pdf [file ppat.1009729.s008.pdf]

Reviewer #1: 1) In the abstract, the authors conclude: “it appears that interactions critical to replication and immune evasion can occur simultaneously with the same molecules of P protein and propose that replication complexes might be directly involved in STAT1 antagonism”. A similar idea is developed in the discussion (after line 569).

I disagree with this idea: N and the ribonucloproteins are essentially present inside the Negri bodies (Nikolic et al. Nat Com. Ref 13) whereas P shuttles between the cytoplasm and the Negri bodies. In the transfection system that the authors have used, the Negri bodies which are the viral factories are not present and there is no viro-induced compartmentalization. This viro-induced compartmentalization, in which genome transcription and replication takes place, is mediated by liquid-liquid phase separation. STAT1 might be excluded from the viral factory (or poorly present in it) (for a discussion on the interplay between viral factories and innate immunity, see the review by Nevers et al. Biochim Biophys Acta Mol Cell Res. 2020 Dec;1867(12):118831). Therefore, it is highly plausible that STAT 1 and N never colocalize in infected cells and thus do not simultaneaously interact with the same P protein.

Therefore, either the authors demonstrate the presence of STAT 1 in the viral factories (i.e. they perform experiments in infected cells) or they discuss the alternative hypothesis at least at the same level as theirs.

We thank the reviewer for raising this important point. We note that previous studies have used microscopy to analyse STAT1 localization in infected cells but have not provided any indications that STAT1 is excluded from Negri bodies. To confirm this, we immunostained infected cells for pY-STAT1, confirming no evidence of exclusion. Thus, it appears likely that STAT1 can localize with Negri bodies, consistent with a lack of exclusion due to either liquid-liquid phase separation or competitive binding with N-RNA. Thus, STAT1 may colocalize with N-RNA/P protein such that replication complexes may contribute to STAT1 antagonism. Based on the reviewer’s comments, we have included these new data and amended the text clarifying on the localization of pY-STAT1 and its potential implications, and to highlight that precise roles of replication complexes in STAT1 antagonism (or potentially other roles of STAT1 in replication) are hypothesised and await further research. We also note that while viral inclusions may function to sequester replication complexes from cellular detection (e.g. pattern recognition proteins) as suggested in the interesting review cited (and now included as ref. 54 in the ms.), STAT1/other STATs have been reported localized to replication compartments including N protein rich compartments of several other mononegaviruses, and Negri Bodies in fact incorporate TLR3 indicative of a complex relationship. We have included brief discussion of these points, together with relevant references, and thank the reviewer for encouraging us to consider these points. The following edits have been made:

Lines 98-101 of the abstract: Thus, it appears that interactions critical to replication and immune evasion can occur simultaneously with the same molecules of P protein so that binding of P protein to activated STAT1 can potentially occur without interrupting interactions involved in replication. These data suggest that replication complexes might be directly involved in STAT1 antagonism.

We rewrote the author’s summary and in response to this reviewer’s comment we edited to say (lines 112-115) These data suggest that P-protein-STAT1 interaction, critical to immune evasion, can occur without interrupting interactions underlying replication, and so replication complexes might be directly involved in STAT1 antagonism.

The final sentence of the introduction. Line 212-215 “Thus, binding of STAT1 to P protein for immune evasion appears not to disrupt interactions with N protein involved in replication, suggesting that replication complexes may contribute to STAT1 antagonism.”

We have added to the results describing the new experiments in infected cells. The text is Lines 513-539 starting "In RABV-infected cell, N protein and associated RNA, P protein and L protein accumulate in Negri body compartments….."

We have edited the Discussion to include these results, lines 623-632. "Whichever model is correct awaits further structural definition, but clearly the data indicated that P protein-STAT1 interaction for immune evasion does not interfere with interactions of P protein with N-RNA critical for replication, or *vice versa*; indeed, pY-STAT1 was not excluded from Negri bodies in infected cells, consistent with a lack of competitive binding. Together these data suggest that replication complexes may participate directly in the antagonism of STAT1. This structural organization of the P_{CTD} would therefore appear to provide a novel and efficient interaction hub at the interface of active replication and antagonism of the resulting antiviral response. It is also possible that the capacity of N-RNA/P to interact with STAT1 results in recruitment of STAT1 with some positive role in viral replication. Consistent with this, STATs can have ambiguous (pro- or antiviral) roles in infection [62]. Delineation of these possibilities is the focus of further research."

Methods on lines 750-754 and 786-796 include assays of the infected cells.

2) It is a bit disappointing that the mutants (with the exception of S389E) have only been constructed in the context of N-pep and not in the context of its cyclized version, as the latter is clearly more relevant. Nevertheless, I understand that it can take a long time to produce and characterize all the mutants in the cyclized version.

Reviewer #2: One major concern is that the homology modeling and molecular dynamics simulation were performed with undecamer of N, which is an artificial oligomer. In the virion, N is known to begin with a decameric ring of N, with gradual increasing radius, reaching a final helical parameter of about 38-39 N/turn in the trunk part of the bullet (Ge et al., Science 2010). Though the location of L attachment by P in the virion is not defined as yet, it can be ruled out to be near the tip (the decameric ring) part of the bullet. The orientation of N is drastically different in the undecamer ring as compared to N in the trunk part of the virion. The authors should repeat these computational work with N oligomers properly oriented as in the trunk, perhaps with a box containing 4x4 N subunits.

Thank you very much for your suggestion, while a time-consuming process to remodel and re-perform the MD simulations, we remodelled the docking and MD similuations with this suggestion which provides a better understanding of the real-world scenario interaction between N and P-proteins. We hope our model could give valuable suggestions for those working in the field. The following edits to the text have been made:

Lines 165-173 of the introduction now describe both the ring and virion structures. "Available crystal structures of the rabies N protein in….."

Figure 4 presents the new model showing the predicted contacts are retained.

Methods in lines 803 to 805 have been edited to describe the new model.

Part III – Minor Issues: Editorial and Data Presentation Modifications
Please use this section for editorial suggestions as well as relatively minor modifications of existing data that would enhance clarity.

Reviewer #1: The reference 47 (Green and Luo, PNAS) is mentioned in the list of references (it is indeed a pertinent one). However, I cannot find where it is quoted in the text.

Line 404 it is now reference 49 and it is in the grouping [47-50] discussing conformational change/fold on P/N interaction.

Reviewer #2: (No Response)
